# Supplementary material for: Transgenerational dynamics of rDNA copy number in Drosophila male germline stem cells
Source: eLife. 2018 Feb 13;7:e32421. doi: 10.7554/eLife.32421 (PMC5811208; doi:10.7554/eLife.32421)
Supplement: Supplementary file 1. [file elife-32421-supp1.docx]

**Supplementary Table 1. rRNA SNP probes**

SNP between X and Y are indicated in red. The areas masked by the masking oligos are indicated by underlines.

| **Probe Set** | **5’-Sequence-3’-Quasar 570/670** |  |
| --- | --- | --- |
| X SNP1 | AAAAAATACAAGTATTTAATCACATA |  |
| Y SNP1 | AAAAGATACAAGTATTTAATCACATA |  |
| SNP1 Mask | TATGTGATTAAATACT |  |
|  |  |  |
| X SNP2 | GTTTCTTCGATTTTCATGTTCGAAAC |  |
| Y SNP2 | GTTTTTTCGATTTTCATGTTCGAAAC |  |
| SNP2 Mask | GTTTCGAACATGAAAA |  |
|  |  |  |
| X SNP3 | AAATATTTATTAACGGTAAGGATATT |  |
| Y SNP3 | AAATGTTTATTAACGGTAAGGATATT |  |
| SNP3 Mask | AATATCCTTACCGTTA |  |
|  |  |  |
| X SNP4 | TTAGGCATTTTTGTTTTACTTGAAAA |  |
| Y SNP4 | TTAGCCATTTTTGTTTTACTTGAAAA |  |
| SNP4 Mask | TTTTCAAGTAAAACAA |  |
