# Supplementary material for: Transgenerational dynamics of rDNA copy number in Drosophila male germline stem cells
Source: eLife. 2018 Feb 13;7:e32421. doi: 10.7554/eLife.32421 (PMC5811208; doi:10.7554/eLife.32421)
Supplement: Supplementary file 2. [file elife-32421-supp2.docx]

**Supplementary Table 2: qPCR primers**

| **Primer Name** | **5’-Sequence-3’** | **Reference** |
| --- | --- | --- |
| tRNA-K-CTT-qF | CTAGCTCAGTCGGTAGAGCATGA | (Aldrich and Maggert, 2014; Paredes and Maggert, 2009b) |
| tRNA-K-CTT-qR | CCAACGTGGGGCTCGAAC |  |
| 18S-qF | AGCCTGAGAAACGGCTACCA |  |
| 18S-qR | AGCTGGGAGTGGGTAATTTACG |  |
| 28S-qF | AATGGATGTGATGCCAATGTA | (Greil and Ahmad, 2012) |
| 28S-qR | TTCAGTGGATCGCAGTATGG |  |
| 5.8S-qF | GCTCATGGGTCGATGAAGAA | This study |
| 5.8S-qR | GGACTGCGATATGCGTTCA |  |
| Gapdh-qF | TAAATTCGACTCGACTCACGGT | DRSC FlyPrimerBank |
| Gapdh-qR | CTCCACCACATACTCGGCTC |  |
| R1 qPCR2 fw | TAGAGCTTGTAGTGGTCGAG | This study |
| R1 qPCR2 rv | ATGGGTCGTCGGCATGATCT |  |
| R2 qPCR fw | ATGAACTGTTATCGCGTCCG |  |
| R2 qPCR rv | AAACGCGTGGGTTGACTAAC |  |
