# Supplementary material for: Transgenerational dynamics of rDNA copy number in Drosophila male germline stem cells
Source: eLife. 2018 Feb 13;7:e32421. doi: 10.7554/eLife.32421 (PMC5811208; doi:10.7554/eLife.32421)
Supplement: Supplementary file 3. [file elife-32421-supp3.docx]

**Supplementary Table 3. Probes for rDNA FISH**

| **Probe Target** | **5’-Sequence-3’** |
| --- | --- |
| Y chromosome | Cy-5-(AATAAAC)_6_ |
| 240-bp IGS | Alexa488-TCCATTCACTAAAATGGCTTTTCTCTATAATACTTA  GAGAATATGGGAATATTTCAACATTTTTCACT |
| 18S rDNA probe | Stellaris probes. Each oligo was labelled with Quasar 570 on 3’ side.  Tataactactggcaggatcaac, catggcttaatctttgagacaa, tcacttttaattcgtgtgtact, actgatataatgagccttttgc, ctgttaacgatctaaggaacca, agaattaccacagttatccaag, aggttcatgttttaattgcatg, tagcctaataaaagcacacgtc, aatataacgatcttgcgatcgc, atacgatctgcatgttatctag, acatttgaaagatctgtcgtcg, gtcctagatactaccatcaaaa, gatatgagtcctgtattgttat, agtgtactcattccaattacag, caattggtccttgttaaaggat, ccgcaacaactttaatatacgc, agcacaagttcaactacgaacg, acaattgtaagttgtactaccc, atataagaactccaccggtaat, tgcaggtttttaaataggagga, cccacaataacactcgtttaag, tgctttaagcactctaatttgt, cacagaatattcaggcatttga, cagaacagaggtcttatttcat, cctcttgatctgaaaaccaatg, ccaaactgcttctattaatcat, ttaagttagtcttacgacggtc, aacatctttggcaaatgctttc, ctctaactttcgttcttgatta, tcgtttatggttagaactaggg, gagagagccataaaagtagcta, aattcctttaagtttcagcttt, aatctgtcttacacacttatgt, ccatagattcgagaaagagcta, atcactccacgaactaagaacg, ttcgttatcggaattaaccaga, caccataatcctgaagatatct, gaatgaaggctacataagcttc, acacaataagcattttactgcc, gctccacttacataaacacatt, gtgtccttataatgggacaaac, gcaatttgtccatttaagaagc, ctgttattgctcaatctcatta, ggtctaggaaatacacgttgat, ttcacaatcccaagcatgaaag, gaattccaagttcatcgtgaac, caatgcgagttaatgactcaca, taattcaatcggtagtagcgac |
